# Association of *MICA *with rheumatoid arthritis independent of known *HLA-DRB1 *risk alleles in a family-based and a case control study

**DOI:** 10.1186/ar2683

**Published:** 2009-05-01

**Authors:** Holger Kirsten, Elisabeth Petit-Teixeira, Markus Scholz, Dirk Hasenclever, Helene Hantmann, Dirk Heider, Ulf Wagner, Ulrich Sack, Vitor Hugo Teixeira, Bernard Prum, Jana Burkhardt, Céline Pierlot, Frank Emmrich, François Cornelis, Peter Ahnert

**Affiliations:** 1 Center for Biotechnology and Biomedicine (BBZ), University of Leipzig, Deutscher Platz 5, 04103 Leipzig, Germany; 2 Translational Centre for Regenerative Medicine, University of Leipzig, Philipp-Rosenthal-Str. 55, 04103 Leipzig, Germany; 3 Fraunhofer Institute for Cell Therapy and Immunology IZI, Perlickstr. 1, 04103 Leipzig, Germany; 4 GenHotel-EA3886, Evry-Paris VII Universities, 2 rue Gaston Crémieux, 91057 Evry-Genopole cedex, France; 5 Institute for Medical Informatics, Statistics and Epidemiology, University of Leipzig, Härtelstr. 16-18, 04107 Leipzig, Germany; 6 Health Economics Research Unit, Department of Psychiatry, University of Leipzig, Liebigstr. 26, 04103 Leipzig, Germany; 7 Medical Clinic and Polyclinic IV, University Hospital Leipzig, Liebigstr. 22, 04103 Leipzig, Germany; 8 Institute of Clinical Immunology and Transfusion Medicine, University of Leipzig, Johannisallee 30, 04103 Leipzig, Germany; 9 Faculty of Medicine, University of Coimbra, Rua Larga, 3004-504 Coimbra, Portugal; 10 Statistics and Genome laboratory, La genopole, 523 place des Terrasses, 91000 Evry, France; 11 Hôpital Sud Francilien, 59 Boulevard Henri Dunant, 91106 Corbeil-Essonnes cedex, France; 12 Hôpital Lariboisière, AP-HP, 2 rue Ambroise – Paré, 75475, Paris cedex 10, France

## Abstract

**Introduction:**

The gene *MICA *encodes the protein major histocompatibility complex class I polypeptide-related sequence A. It is expressed in synovium of patients with rheumatoid arthritis (RA) and its implication in autoimmunity is discussed. We analyzed the association of genetic variants of *MICA *with susceptibility to RA.

**Methods:**

Initially, 300 French Caucasian individuals belonging to 100 RA trio families were studied. An additional 100 independent RA trio families and a German Caucasian case-control cohort (90/182 individuals) were available for replication. As *MICA *is situated in proximity to known risk alleles of the *HLA-DRB1 *locus, our analysis accounted for linkage disequilibrium either by analyzing the subgroup consisting of parents not carrying *HLA-DRB1 *risk alleles with transmission disequilibrium test (TDT) or by implementing a regression model including all available data. Analysis included a microsatellite polymorphism (GCT)n and single-nucleotide polymorphisms (SNPs) rs3763288 and rs1051794.

**Results:**

In contrast to the other investigated polymorphisms, the non-synonymously coding SNP *MICA*-250 (rs1051794, Lys196Glu) was strongly associated in the first family cohort (TDT: *P *= 0.014; regression model: odds ratio [OR] 0.46, 95% confidence interval [CI] 0.25 to 0.82, *P *= 0.007). Although the replication family sample showed only a trend, combined family data remained consistent with the hypothesis of *MICA*-250 association independent from shared epitope (SE) alleles (TDT: *P *= 0.027; regression model: OR 0.56, 95% CI 0.38 to 0.83, *P *= 0.003). We also replicated the protective association of *MICA*-250A within a German Caucasian cohort (OR 0.31, 95% CI 0.1 to 0.7, *P *= 0.005; regression model: OR 0.6, 95% CI 0.37 to 0.96, *P *= 0.032). We showed complete linkage disequilibrium of *MICA*-250 (D' = 1, *r^2^*= 1) with the functional *MICA *variant rs1051792 (D' = 1, *r^2^*= 1). As rs1051792 confers differential allelic affinity of MICA to the receptor NKG2D, this provides a possible functional explanation for the observed association.

**Conclusions:**

We present evidence for linkage and association of *MICA*-250 (rs1051794) with RA independent of known *HLA-DRB1 *risk alleles, suggesting *MICA *as an RA susceptibility gene. However, more studies within other populations are necessary to prove the general relevance of this polymorphism for RA.

## Introduction

Rheumatoid arthritis (RA) is a common autoimmune disease characterized by chronic inflammatory changes of joints and inner organs. It is estimated that at least 50% of the risk to develop RA is determined by genetic factors [1]. Considerable efforts have been made to elucidate these genetic factors to better understand the disease. However, even after the advent of genome-wide association studies, only somewhat more than half of the estimated genetic risk for RA has been assigned to specific genetic determinants [2]. There is strong evidence [3-5]. that additional genetic risk factors reside within a genomic region containing the strongest known genetic determinants of RA susceptibility, alleles of the *HLA-DRB1 *gene. Identification of additional risk factors within the *HLA-DRB1 *gene region is complicated by the extraordinarily high local linkage disequilibrium (LD): Standard association analyses of genetic variants in candidate gene and genome-wide association studies are prone to confounding due to LD with *HLA-DRB1 *alleles. Successful identification of additional genetic risk factors in this region needs to account for risk conferred by different *HLA-DRB1 *alleles. Within the shared epitope (SE) hypothesis, *HLA-DRB1 *alleles *0101, *0102, *0401, *0404, *0405, *0408, and *1001 are most commonly reported to be associated with risk for RA in European Caucasians [6]. Recently, a new classification of *HLA-DRB1 *alleles was proposed by du Montcel and colleagues [7], taking into account risk-modifying effects of neighboring amino acids. This classification emerged as especially reproducible and reliable [8].

**Figure 1:**
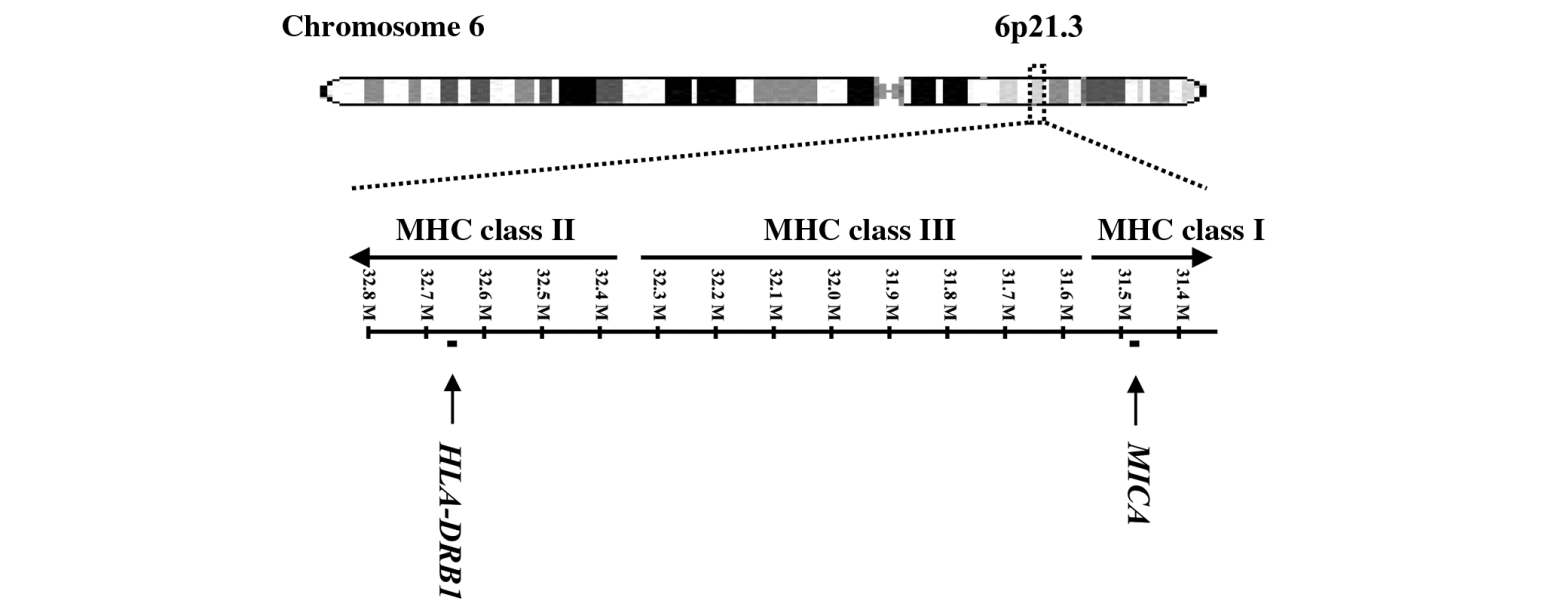
Location of *MICA *relative to the *HLA-DRB1 *locus. Despite a distance of more than one megabase from the rheumatoid arthritis risk factor *HLA-DRB1 *in the major histocompatibility complex (MHC) class II region, there is considerable linkage disequilibrium between markers in both genes. Therefore, *HLA-DRB1 *status must be considered for interpretation of genetic association data.

*MICA *is located within the same genomic region as *HLA-DRB1 *(Figure 1). It encodes the protein major histocompatibility complex class I polypeptide-related sequence A. This protein interacts with the C-type lectin activatory receptor NKG2D (also known as KLRK1) found on natural killer cells, γδ T cells, and certain subgroups of αβ T cells. MICA-NKG2D interaction is believed to be important for eliminating infected or tumorous cells [9]. This interaction is also described to increase inflammatory cytokine production and proliferation of a certain subset of T cells. In consequence, implications in autoimmunity have been discussed [9-12]. MICA is expressed in RA synovium but not in osteoarthritis synovium [12]. Local NKG2D expression is induced by tumor necrosis factor and interleukin-15 [12]. These findings make *MICA *an interesting candidate gene for association studies in RA.

The highly polymorphic gene *MICA *(122 frequency-validated single-nucleotide polymorphisms [SNPs] in SNP database [dbSNP] build 129) was investigated in various RA association studies in different populations. For several SNPs and for a microsatellite marker, associations with protection or risk were shown [4,13-17]. Results for different *MICA *variants were not conclusive but point toward association with RA. Heterogeneity between results of these studies may be due at least partially to confounding of results by LD with *HLA-DRB1 *alleles.

Some studies reported association analyses without controlling for LD of *MICA *with *HLA-DRB1 *alleles at all [14,17]. This makes a conclusion about an independent association of *MICA *intricate. If association analysis is done under the condition of no significant LD between *MICA *and *HLA-DRB1 *alleles [16], the problem prevails: Even weak, non-significant LD may bias *MICA *association analysis since effect sizes of known *HLA-DRB1 *risk alleles are considerably large. Other authors restricted analysis to the patient subgroup without *HLA-DRB1 *risk alleles, ignoring large parts of the data [13]. Alternatively, stratification of data in SE and non-SE subgroups ignores variance of the individual risk of SE alleles within the SE subgroup [15]. In a recent study, case-control pairs were matched 1:1 by *HLA-DRB1 *genotype to control confounding [4]. However, as a disadvantage of this method, large proportions of typical RA patient and control collections are excluded from analysis since certain *HLA-DRB1 *genotypes are common in patients but rare in controls and vice versa.

Our aim was to investigate the role of DNA polymorphisms of *MICA *in French Caucasian RA family trios and in a German Caucasian case-control cohort. Confounding by *HLA-DRB1 *risk alleles was controlled by analysis of the subgroup negative for known *HLA-DRB1 *risk alleles and by logistic regression including all data.

## Materials and methods

### Patients

We analyzed 600 French Caucasian individuals belonging to 200 families grouped in two cohorts of 100 family trios. Characteristics (gender, age at onset, disease duration, erosions, seropositivity, and SE) as well as details on DNA preparation were described previously [18]. Seventy-six percent of French RA index patients were positive for anti-cyclic citrullinated peptide antibodies (CCP^+^). For case-control analysis, 272 German Caucasians were analyzed. Controls were 182 healthy blood donors (mean age ± standard deviation [SD] was 50 ± 7 years, and 80% were female) from the Institute of Transfusion Medicine, University Hospital Leipzig, Germany, and cases were 90 RA patients from the Medical Clinic IV, University Hospital Leipzig, Germany, with the following characteristics: mean age (± SD) at disease onset was 47.1 ± 15.7 years, mean (± SD) disease duration was 26.7 ± 20.5 years, 92% were RA patients seropositive for rheumatoid factor, and 78% were female. All individuals provided informed consent, and the ethics committees of Hôpital Bicêtre (Kremlin-Bicêtre, AP-HP, France) and of the University of Leipzig (Leipzig, Germany) approved the study.

### Genotyping

We investigated three polymorphisms spanning *MICA *for association with RA. For SNP selection, we required frequency validation, a map weight of 1, and a minor allele frequency exceeding 5% in Caucasians. Among 775 SNPs available within the MICA region in Ensemble version 24, 7 were frequency-validated and had a map weight of 1. Within the promoter region, defined as within 5 kb upstream of the start of the gene, we selected *MICA*-300 (rs3763288). According to TESS (Transcription Element Search System) [19], *MICA*-300 co-localizes with a binding site for the transcription factor ETV4. Within the coding region, we selected the non-synonymously coding SNP *MICA*-250 (rs1051794, Lys196Glu) as validation information for this variant was previously published [20,21]. In addition, variant *MICA*-210 (a trinucleotide repeat (GCT)n microsatellite polymorphism within the transmembrane domain) was selected as various associations of this variant with RA were reported previously [15-17,22].

Genotyping was done by applying single-base extension followed by mass spectrometry ('GenoSNIP') as described [23] but with the following modifications: polymerase chain reaction (PCR) and genotyping primers for *MICA*-210: CCTTTTTTTCAGGGAAAGTGC, CCTTACCATCTCCAGAAACTGC [22], and bioCCATGTTTCTGCTG(L)TGCTGCT; *MICA*-300: GGAAGGCTGTGCAGTAATCTAGG, TCCCTTTTCCAGCCTGCC, and bioCTGTGCAGT(L)ATCTAGGCTGAAGG; and *MICA*-250: AAGGTGATGGGTTCGGGAA, TCTAGCAGAATTGGAGGGAG [21], and bioCTCAGGAC(L)ACGCCGGATT. For the *MICA*-250 assay, a genotyping primer bioCTCCAGAG [L]TCAGACCTTGGC, differentiating between a paralogue sequence variant of *MICA *and *MICB*, was genotyped in 558 (63.8%) samples. This assay always indicated amplification of *MICA *and never of *MICB*. PCR products were checked by agarose gel electrophoresis for correct size and sufficient yield. Within the studied population, no Mendel error occurred. No significant departure (*P *≤ 0.05) from Hardy-Weinberg equilibrium was observed in controls (French samples: *P *= 0.240 for non-transmitted chromosomes; German controls: *P *= 0.233; chi-square test with one degree of freedom).

*HLA-DRB1 *was genotyped previously using sequence-specific PCR primers and hybridization of PCR products with probes specific for *HLA-DRB1 *alleles, as described for the French family sample [18] and the German case-control sample [24]. Distribution of *HLA-DRB1 *alleles can be found in the online supplement (Additional data file 1).

### Statistical analysis

For association analysis, we chose a multistep approach. In a first cohort of 100 family trios, selected polymorphisms were tested for association with RA. Those showing nominal association at a significance level of 0.05 or below were tested in a second cohort of 100 French family trios. A decrease in *P *value in the combined French cohorts was taken as strong evidence in favor of association. These polymorphisms were further analyzed in a German Caucasian case-control cohort.

Haplotypes were estimated using the software HAPLORE (HAPLOtype REconstruction) [25]. For these estimations, data of SNPs located between *MICA *and *HLA-DRB1 *were included (rs1800629, rs909253, rs3093553, and rs3093562 for the second French cohort and additionally rs1043618, rs2075800, rs1799964, rs1800630, rs3093662, and rs3093664 for the first French cohort; data available online [26]). We successfully assigned haplotypes for 95% of all families (minimum posterior probability was 90% and mean posterior probability was greater than 99.9%).

Transmission disequilibrium test (TDT) for association and linkage with RA was calculated as described by Spielman and colleagues [27]. For subgroup analyses, the subgroup without *HLA-DRB1 *risk alleles was defined by the absence of SE alleles. This is identical with allele L according to the classification by du Montcel and colleagues [7]. Derived haplotype information allowed identification of transmitted and non-transmitted chromosomes.

For conditional logistic regression analysis of families, LogXact (Cytel Inc., Cambridge, MA, USA) was used. Within this analysis, *HLA-DRB1 *allele classification was according to du Montcel and colleagues [7]. The S3P allele consisted of alleles *0101, *0102, *0404, *0405, *0408, and *1001, and the S2 allele consisted of *0401. We applied the convention that allele L denotes alleles S1, S3D, and X as the associated risk for RA of the latter three alleles was found to be of similar magnitude [7,8]. Of the index patients of all 200 French families, 53% and 45% contributed to allele groups S3P and S2, respectively. Twenty-one percent were homozygous for allele L. In regression analysis, we modeled the transmission probability of a haplotype toward affected children given the competitive haplotype of a parent. This method is known as conditional logistic regression. To include *HLA-DRB1 *alleles in the model, allele L was used as the reference group. To ensure independence of *MICA *association from *HLA-DRB1 *risk alleles, a likelihood ratio test (LRT) was done. Here, the likelihood of the model including *HLA-DRB1 *alleles and *MICA *was compared with a model including *HLA-DRB1 *alleles only. A significant increase of the model's likelihood that includes polymorphism *MICA *(that is, an LRT *P *value of less than 0.05) indicates an association of the *MICA *polymorphism independent of the known association of *HLA-DRB1 *alleles. Analogously, we checked for interactions between *MICA *and *HLA-DRB1*. Additional methodological remarks to this method are given in the online supplement (Additional data file 2).

Within the case-control cohort, haplotyping was not resolvable with the same accuracy as for the family cohorts. Hence, the logistic regression model was based on unphased data of *MICA*-250 and *HLA-DRB1*. It included all case-control individuals, accounting for *HLA-DRB1 *risk alleles. *HLA-DRB1 *classification according to du Montcel and colleagues [7] as described above was applied. Cases of the case-control cohort contributed to allele groups S3P (42%) and S2 (36%). Twenty-one percent were homozygous for allele L. Within the model, genotypes were coded (0, 1, and 2), with 2 coding for the homozygous minor allele. Thus, an additive model was implemented. LRTs were done similarly to the conditional logistic regression model described above. Multimarker LD analysis was done using the software MIDAS (Multiallelic Interallelic Disequilibrium Analysis Software) [28]. For the exact Mantel-Haenszel test, the software StatsDirect was used [29]. If not indicated otherwise, *P *values were not corrected for multiple testing.

## Results

### Association of *MICA *with rheumatoid arthritis within the first French family cohort

We analyzed three polymorphisms within the gene *MICA*: *MICA*-300 (rs3763288) within the 5' region of the gene (promoter region), *MICA*-210 (trinucleotide repeat (GCT)n microsatellite polymorphism within the transmembrane domain), and *MICA*-250 (non-synonymously coding SNP, rs1051794, Lys196Glu).

In standard analysis (TDT without accounting for linkage with *HLA-DRB1*), we found significant undertransmission of *MICA*-250A in the first French family cohort (Table 1a). Our first strategy to account for potential LD with *HLA-DRB1 *was to restrict analysis to parents negative for *HLA-DRB1 *risk alleles. Here, we also found protective association of *MICA*-250A and RA (Table 1b). In our second strategy, we controlled for LD with *HLA-DRB1 *risk alleles by conditional logistic regression. *MICA*-250A again emerged as a protective factor as haplotypes including *MICA*-250A were significantly undertransmitted to affected children. The LRT was significant, demonstrating that *MICA*-250 is associated with RA independent of known *HLA-DRB1 *risk alleles (Table 1c).

**Table 1:** Association of *MICA *polymorphisms within the first French Caucasian family cohort

	*MICA*-210					*MICA*-250	*MICA*-300
			
(a) French population 1 – all individuals without controlling for LD with *HLA-DRB1*							
Minor allele	4	5	5.1	6	9	A	A
Frequency in cases/controls^a^	7%/12%	12%/7%	42%/36%	26%/25%	13%/20%	23%/34%	8%/4%
Minor allele transmitted/untransmitted	13/21	21/13	44/36	35/33	17/29	26/48	15/7
Transmission rate	38%	62%	55%	51%	37%	35%	68%
TDT *P *value	0.172	0.172	0.376	0.815	0.080	0.011	0.091
							
(b) French population 1, subgroup without *HLA-DRB1 *risk alleles							
Minor allele transmitted/untransmitted	5/10	5/4	18/15	18/12	5/10	6/18	3/1
Transmission rate	33%	56%	55%	60%	33%	25%	75%
TDT *P *value	0.200	0.740	0.600	0.270	0.200	0.014	0.317
							
(c) French population 1, all individuals, controlling for LD with *HLA-DRB1 *by conditional logistic regression							
OR (95% CI)^b^	0.59 (0.25–1.34)	1.48 (0.64–3.54)	1.28 (0.77–2.16)	1.23 (0.71–2.17)	0.51 (0.24–1.05)	0.46 (0.25–0.82)	1.2 (0.37–4.15)
*P *value	0.235	0.428	0.379	0.518	0.072	0.007^d^	0.944
LRT^c ^*P *value	0.165	0.319	0.314	0.433	0.048	0.005^d^	0.728

^a^Controls are non-transmitted alleles; ^b^odds ratio of transmission of minor allele versus transmission of major allele as determined in logistic regression; ^c^likelihood ratio test evaluating model including *HLA-DRB1 *alleles S2 and S3P and *MICA*-250 versus S2 and S3P only. For the *HLA-DRB1 *locus, allele L was used as reference. ^d^*P *value corrected for multiple testing less than 0.05. CI, confidence interval; LD, linkage disequilibrium; TDT, transmission disequilibrium test.

Association of *MICA*-250 with RA was stronger compared with association of other analyzed single markers (Table 1) and with three-marker haplotypes consisting of *MICA*-300, *MICA*-250, and *MICA*-210 (data not shown). Therefore, only *MICA*-250 was included in further validation studies within a second independent French Caucasian family cohort and a case-control cohort of German Caucasian origin.

### Association analysis within the second and combined first and second French family cohorts

Within the second French family cohort, we found the same trend for protective association of *MICA*-250A with RA in standard analysis and in both the *HLA-DRB1 *risk allele-negative subgroup analysis and conditional logistic regression (Table 2). In combined analysis of both French family cohorts, association of *MICA*-250 was comparable with the association in the first French family cohort in standard analysis and in analysis of the subgroup negative for *HLA-DRB1 *risk alleles. In conditional logistic regression analysis, association in the combined cohorts was even more significant than in the first cohort alone (Tables 1c and 2c). Additionally, conditional logistic regression was done with a model in which S3P alleles were differentiated into three groups as described [8], accounting for potential differences in risk of these three groups for RA. Within this analysis, 79, 56, and 15 individuals contributed to the S3P*01, S3P*04, and S3P*10 alleles, respectively. This analysis gave similar results (data not shown). Interactions between *MICA*-250 and *HLA-DRB1 *alleles were not significant (data not shown). Full details of the regression model are shown in the online supplement (Additional data file 3). When the analysis of the combined first and second French cohorts was restricted to CCP^+ ^RA, the protective association with *MICA*-250 A was also found (odds ratio [OR] 0.53, 95% confidence interval [CI] 0.33 to 0.83, *P *= 0.005; LRT *P *value = 0.003).

**Table 2:** Association of *MICA *polymorphism within the second and combined first and second French Caucasian family cohort

	2nd French family cohort	1st + 2nd French family cohort
(a) All individuals without controlling for LD with *HLA-DRB1*		
Minor allele	A	A
Frequency in cases/controls^a^	27%/32%	25%/33%
Minor allele transmitted/untransmitted	37/46	63/94
Transmission rate	45%	40%
TDT *P *value	0.328	0.015
		
(b) Subgroup without *HLA-DRB1 *risk alleles		
Minor allele transmitted/untransmitted	12/16	18/34
Transmission rate	43%	35%
TDT *P *value	0.450	0.027
		
(c) All individuals, controlling for LD with *HLA-DRB1 *by conditional logistic regression		
OR (95% CI)^b^	0.68 (0.4–1.15)	0.56 (0.38–0.83)
*P *value	0.158	0.003
LRT^c ^*P *value	0.122	0.002

^a^Controls are non-transmitted alleles; ^b^odds ratio of transmission of minor allele versus transmission of major allele as determined in logistic regression; ^c^likelihood ratio test evaluating model including *HLA-DRB1 *alleles S2 and S3P and *MICA*-250 versus S2 and S3P only. For the *HLA-DRB1 *locus, allele L was used as reference. Effects of S2 and S3P alleles are presented in the online supplement (Additional data file 3). CI, confidence interval; LD, linkage disequilibrium; TDT, transmission disequilibrium test.

### Association analysis within the case-control cohort

After demonstrating association of *MICA *with RA in French Caucasian family trios and its independence from HLA risk alleles, we analyzed the effect of *MICA *within a German Caucasian case-control cohort. Frequencies of *MICA*-250A were similar within the German and French populations (33% in controls). Again, we found protective association of *MICA*-250A with RA in standard analysis and within the subgroup of the case-control cohort not carrying SE alleles (Tables 3a and 3b). Logistic regression including all individuals demonstrated a significant protective effect as well. Significance in the LRT showed that this association was independent of *HLA-DRB1 *risk alleles (Table 3c). Details of the regression model are given in the online supplement (Additional data file 4). Additionally, conditional logistic regression was done with a model in which S3P alleles were differentiated into three groups (S3P*01, S3P*04, and S3P*10) as described [8], accounting for potential differences of these three groups in risk for RA. This analysis resulted in similar results (data not shown).

### Analysis of linkage disequilibrium

LD was analyzed within parents of the family cohorts and in the case-control cohort. As the German cohort was smaller, power to detect LD was decreased compared with power to detect LD within the French cohorts. Significant LD was found between *HLA-DRB1*-S3P and *MICA*-250A within parents of the French family cohorts (D' = +0.21, *P *< 0.001). Interestingly, this LD was positive between *HLA-DRB1 *risk alleles of subgroup S3P and the protective allele *MICA*-250A. In-depth analysis of the S3P group revealed that this resulted mainly from LD between HLA-DRB1*01 and *MICA*-250A, which was significant within parents of the family cohorts and cases from the case-control cohort (D' = +0.38 and +0.25 with *P *values of 2 × 10^-7 ^and 0.047, respectively). Significant negative LD was found between *HLA-DRB1*-S2 and *MICA*-250A (D' = -0.51, *P *< 0.01) in French parents. No significant LD was found between *HLA-DRB1*-L within the family cohorts and individuals of the case-control cohort. In consequence, there was no significant correlation of carriage of *MICA*-250A with carriage of positive or negative SE status. LD was also analyzed between *MICA*-250 and rs1051792, another coding SNP with functional implications [30]. Within a representative sample of 182 French Caucasian and 181 German Caucasian cases and controls, both polymorphisms were in perfect LD (*r^2^*= 1, D' = 1).

### Representation of association analysis in all informative families controlling for linkage disequilibrium with HLA-DRB1

An advantage of the conditional logistic regression approach is the integration of all data from all informative parents with respect to *HLA-DRB1 *and *MICA*. A single statistic reveals independent association of *MICA*-250. However, it is of interest to compare subgroup analysis of parents negative for *HLA-DRB1 *risk alleles with results of the regression model analyzing all data in detail (Tables 2b and 2c). A major difference is that the regression model additionally includes information of parents that are informative (that is, heterozygous) for *MICA *and that are also heterozygous for *HLA-DRB1 *risk alleles. How can the effect of *MICA*-250 on transmission be represented within these parents, devoid of the effect of *HLA-DRB1 *risk alleles? We propose to stratify *HLA-DRB1 *heterozygous parents according to their genotype. The transmission ratio under the null hypothesis of no association within these parents will differ from a 50/50 ratio reflecting the different risk levels of both *HLA-DRB1 *alleles. However, under the null hypothesis of no association of *MICA*-250, a two-marker haplotype consisting of *MICA*-250A and a certain *HLA-DRB1 *allele should have the same transmission rate as a two-marker haplotype consisting of *MICA*-250G and the same *HLA-DRB1 *allele. A deviation from this transmission rate represents an independent effect of *MICA*-250A quantifiable as an OR of *MICA*-250A transmission. As we applied the classification of du Montcel and colleagues [7] of *HLA-DRB1 *alleles, three different independent strata of *HLA-DRB1 *heterozygote parents exist: S3P/S2, S2/L, and S3P/L. Within all of these strata, we always found a decreased transmission of haplotypes carrying *MICA*-250A compared with the respective haplotype carrying *MICA*-250G (OR 0.33, 95% CI 0.02 to 5.11; OR 0.45, 95% CI 0.04 to 6.76; and OR 0.44, 95% CI 0.04 to 2.73, respectively, data of all families) (Additional data file 5). These observations are consistent with the significant protective association of *MICA*-250A revealed by conditional logistic regression (Table 2).

When we additionally include data from parents homozygous for *HLA-DRB1*, we can analyze the OR of *MICA*-250A on transmission within these parents when we compare the observed transmission ratio of *MICA*-250A versus the expected transmission ratio (Additional data file 5). The expected transmission ratio is 50/50 (transmitted/non-transmitted) within these parents under the null hypothesis of no effect of *MICA*-250A. We now can combine information from all parents informative for *MICA*-250 by combining all four ORs of all four independent strata with exact Mantel-Haenszel methodology. This analysis confirmed a significant undertransmission of *MICA*-250A within all data of all families (OR 0.48, 95% CI 0.25 to 0.91, *P *= 0.02, Fisher exact test).

## Discussion

The aim of this study was to analyze the association of polymorphisms of *MICA *with risk for RA while controlling for the effects of *HLA-DRB1 *risk alleles. We successfully identified *MICA*-250A as a new independent marker associated with protection from RA susceptibility. We analyzed the association of three genetic variants of the gene *MICA *with susceptibility to RA in a French Caucasian family cohort. In validation studies (including an additional independent French Caucasian family cohort and a German Caucasian case-control cohort), we focused on the non-synonymously coding SNP *MICA*-250 (rs1051794, Lys196Glu). In our first French family cohort, this SNP presented with the strongest evidence for association in terms of *P *values and transmission rate (Table 1). Association of three-marker haplotypes of *MICA *with RA was not statistically significant. Therefore, we did not investigate haplotype association further. However, it cannot be excluded that association of *MICA*-250 with RA may be related to an unknown allelic variant in linkage with these haplotypes as haplotypes were inferred and have error margins.

Within all combined French families, we found a significant undertransmission of *MICA*-250A in the TDT (Table 2). Therefore, we hereby provide evidence for linkage and association of *MICA*-250A with RA. This transmission analysis within trio families would not be affected by hidden population stratification. The association was also evident in conditional logistic regression analyses including all parents informative for *MICA*-250A and controlling for LD with *HLA-DRB1 *risk alleles (Table 2c). We did not find any indication that the observed protective effect of *MICA*-250A is especially present on the background of certain *HLA-DRB1 *alleles as interaction analyses of *MICA*-250 and *HLA-DRB1 *alleles in the regression model did not result in a significantly increased likelihood (data not shown). Additionally, detailed transmission analysis of *MICA*-250 within parents heterozygous or homozygous for *HLA-DRB1 *always resulted in a protective effect of *MICA*-250A of comparable magnitude irrespective of present *HLA-DRB1 *alleles (Additional data file 5). Analysis of the CCP^+ ^subset showed that *MICA*-250 also associates with CCP^+ ^RA. We confirmed the protective effect in a German Caucasian RA case-control cohort (Table 3), which indicates that the protective effect may not be restricted to the French Caucasian population alone.

**Table 3:** Case-control analysis in German Caucasians

(a) All individuals without controlling for LD with *HLA-DRB1*	
Minor allele	250A
Allele frequency in cases/controls	22%/33%
Total alleles of RA cases/controls	178/368
OR (95% CI)	0.60 (0.4–0.9)
OR *P *value	0.016
(b) Subgroup without *HLA-DRB1 *risk alleles	
Frequency in cases/controls	14%/34%
Total alleles of RA cases/controls	50/216
OR (95% CI)	0.31 (0.1–0.7)
OR *P *value	0.005
(c) All individuals, controlling for LD with *HLA-DRB1 *by logistic regression	
OR (95% CI)	0.6 (0.37–0.96)
*P *value	0.032
LRT^a ^*P *value	0.022

^a^Likelihood ratio test evaluating model including *HLA-DRB1 *alleles S2 and S3P and *MICA*-250 versus S2 and S3P only. For the *HLA-DRB1 *locus, allele L was used as reference. CI, confidence interval; LD, linkage disequilibrium; OR, odds ratio; RA, rheumatoid arthritis.

True association of *MICA*-250 with RA may be either feigned or masked by LD with known risk alleles. Therefore, we controlled for the separate contributions of *MICA*-250 and *HLA-DRB1 *alleles (S3P, S2, and L) to the observed effect by logistic regression. This allowed us to make use of data from all patients. However, it could be argued that this logistic regression might be affected by stratification of the individual *HLA-DRB1 *risk alleles in the groups used in the model. Hence, we also analyzed the subgroup of patients not carrying *HLA-DRB1 *risk alleles. Naturally, this subgroup does not contain data from all patients, but results are completely independent from the excluded *HLA-DRB1 *risk alleles. Both methods showed association of *MICA*-250A with RA.

In this context, it is of interest that, within all genome-wide association studies of RA published thus far, *MICA*-250 was found to be nominally associated: *MICA*-250A had a protective effect (OR 0.82, 95% CI 0.73 to 0.92, *P *= 0.0008, not corrected for genome-wide testing) within CCP^+ ^RA in North American samples [31]. Similar findings result from a genome-wide study in a British RA cohort, in which data for an SNP in perfect LD with *MICA*-250 are available (rs1051792: OR 0.85, 95% CI 0.77 to 0.93, *P *= 0.0008, not corrected for genome-wide testing) [32]. These findings corroborate our observation of a protective effect of *MICA*-250A in CCP^+ ^RA. *MICA*-250 was also associated with RA in a genome-wide study in a Spanish Caucasian cohort (*P *= 0.02, not corrected for genome-wide testing) [33]. In these genome-wide studies, association analysis was reported without controlling for LD of *MICA *alleles with *HLA-DRB1 *alleles. If LD structure in Caucasians in these genome-wide studies was similar to that in our study (that is, if positive LD between HLA-DRB1*0101 and *MICA*-250A was present), LD-corrected protective association of *MICA*-250A would be even stronger than reported.

The microsatellite polymorphism *MICA*-210 was studied in different populations. In Spanish [15] and Canadian [17] Caucasians, a protective effect was seen for allele *MICA*-210 6.0, whereas in Korean Asians [16], a protective effect was seen for *MICA*-210 9.0. No association of *MICA*-210 was seen in another Spanish Caucasian RA study [14]. None of these studies additionally analyzed *MICA*-250. However, in our study, strong LD between *MICA*-210 9.0 and *MICA*-250A was found (D' = 0.98, *P *< 10^-15^). Therefore, previous findings in Koreans are in accordance with our results. It is of interest that in this study only a single *HLA-DRB1 *RA susceptibility allele (*0405) predominates and no LD was found with *0405 and *MICA*-210 9.0, so that association analysis was hardly influenced by linkage with known *HLA-DRB1 *risk alleles. This is different from the Caucasian studies of the (GCT)n polymorphism: In our data, we found considerable LD between various *MICA*-210 alleles and *HLA-DRB1 *risk alleles (data not shown). We might speculate that complex LD structure between *MICA*-210 alleles and *HLA-DRB1 *alleles may at least partially explain differing results in Caucasian association studies of *MICA*-210 and RA. This is especially relevant as these studies either did not account at all or only partially accounted for LD with *HLA-DRB1 *alleles.

In recently published work, *HLA-DRB1*-matched cases and controls were analyzed mainly in American Caucasians in order to identify genetic factors associated with CCP^+ ^RA in addition to known *HLA-DRB1 *risk alleles [4]. Within the *MICA *genomic region, significant evidence for independent association with RA was found with a maximum association within HLA-C. This association was attributed to the risk of the A1-B8-DRB1*03 haplotype. Additionally, haplotypes carrying HLA-DRB1*0404 were described to be *HLA-DRB1*-independent risk factors. An analysis of *MICA*-250 was not reported in this study. There is evidence that association of *MICA*-250A in our data represents an additional disease-modifying factor, independent of described risk factors in the American Caucasian study. This evidence results from the observation that a protective association of *MICA*-250A is still observed when all parents carrying either HLA-DRB1*03 or HLA-DRB1*0404 were excluded (OR 0.56, 95% CI 0.35 to 0.89, *P *= 0.013; LRT *P *value = 0.009).

Generally, an observed association of a polymorphism with a phenotype need not arise from a direct functional effect of this polymorphism. It may simply originate from LD with a functional polymorphism. Therefore, it is of interest that the amino acid change due to *MICA*-250A (Lys196Glu) is predicted to influence Hsp70 binding [34]. Possibly even more relevant, SNP rs1051792, in perfect LD with *MICA*-250 in Caucasian HapMap data and in our data, was experimentally shown to influence binding of the NKG2D receptor [30]. Variant rs1051792A, corresponding to *MICA*-250A, was shown to strongly bind NKG2D. All other alleles lead to weaker binding. Several studies show that NKG2D expression is modulated by MICA expression level with consequences for immune reactions. Wiemann and colleagues [35] showed that persistent expression of MICA in transgenic mice resulted in downregulation of the amount of surface NKG2D. As a consequence, impaired immune reaction against bacteria and MICA-expressing tumors was observed. In a different context, Mincheva-Nilsson and colleagues [36] observed elevated levels of soluble MICA/MICB and a decreased level of NKG2D within maternal blood of healthy pregnant women. The authors showed that soluble MICA/MICB downregulates NKG2D levels and immune reactions [36]. Therefore, we speculate that an increased affinity of MICA to NKG2D, as must be present in carriers of *MICA*-250A, may have similar effects as increased expression of MICA, resulting in decreased NKG2D expression levels.

In this context, the observation of RA remission during pregnancy may be of interest [37]. Apparently, decrease of NKG2D plays a central role in decreased immune response. During pregnancy, this seems to be triggered by increased levels of MICA/MICB and appears to contribute to tolerance against the fetus and disease remission in women with RA. As pregnant women show both downregulation of NKG2D due to increased MICA expression and remission of RA, it can be speculated that there may be a functional link between these two observations. If *MICA*-250A reports on stronger binding of MICA and if this also results in downregulation of NKG2D levels, this would be consistent with the observed protective effect of *MICA*-250A in our data. As there are many links between the innate and adaptive immune systems and involvement of pathogens in the initiation of RA is discussed (reviewed by Falgarone and colleagues [38]), differences in NKG2D levels induced by functional variants of MICA are not unlikely to have consequences for RA etiology.

## Conclusions

In summary, we present evidence for linkage and association of *MICA*-250 (rs1051794) with RA independently of known *HLA-DRB1 *association in French Caucasians and evidence for association in a German Caucasian population, suggesting *MICA *as an RA susceptibility gene. The association might be explained by functional evidence of rs1051792, an SNP in perfect LD with *MICA*-250. However, more studies within other populations are necessary to prove the general relevance of this polymorphism with RA.

## Abbreviations

CCP^+^: positive for anti-cyclic citrullinated peptide antibodies; CI: confidence interval; LD: linkage disequilibrium; LRT: likelihood ratio test; OR: odds ratio; PCR: polymerase chain reaction; RA: rheumatoid arthritis; SD: standard deviation; SE: shared epitope; SNP: single-nucleotide polymorphism; TDT: transmission disequilibrium test.

## Competing interests

The authors declare that they have no competing interests.

## Authors' contributions

HK helped to carry out the molecular genetic studies, performed acquisition of the data, helped to perform analysis and interpretation of the data, and drafted the manuscript. HH and JB helped to carry out the molecular genetic studies. MS, DHe, BP, CP, EP-T, and FC helped to perform analysis and interpretation of the data. DHa and PA helped to perform analysis and interpretation of the data and to draft the manuscript. VHT and UW (and the European Consortium on Rheumatoid Arthritis Families) contributed to the recruitment of families and to the acquisition of clinical data. FE and US helped to draft the manuscript. All authors read and approved the final manuscript.

## Supplementary Material

Additional data file 1 A table listing the distribution of *HLA-DRB1 *alleles in the analyzed RA cohorts. Click here for file

Additional data file 2 Background information for the conditional logistic regression method applied for family based analysis. Click here for file

Additional data file 3 A table providing detailed results of conditional logistic regression models of all French families. Click here for file

Additional data file 4 A table providing detailed results of logistic regression models of the German case control cohort. Click here for file

Additional data file 5 A table providing a representation of association analysis in all informative families controlling for LD with DRB1. Click here for file
