# Supplementary material for: Association of MICA with rheumatoid arthritis independent of known HLA-DRB1 risk alleles in a family-based and a case control study
Source: Arthritis Res Ther. 2009 May 1;11(3):R60. doi: 10.1186/ar2683 (PMC2714103; doi:10.1186/ar2683)
Supplement: Additional data file 1 — A table listing the distribution of HLA-DRB1 alleles in the analyzed RA cohorts. [file ar2683-S1.pdf]

Additional data file 1: Distribution of *HLA-DRB1* alleles in the analyzed RA cohorts

a)

***HLA-DRB1* allele**	**First French cohort**	**Second French cohort**
DRB1*0101	16.0%	9.5%
DRB1*0102	1.0%	1.0%
DRB1*01other+	1.0%	0.5%
DRB1*03	1.0%	8.5%
DRB1*0401	23.0%	28.0%
DRB1*0402	0.5%	0.0%
DRB1*0403	1.0%	0.0%
DRB1*0404	4.5%	6.5%
DRB1*0405	4.0%	3.0%
DRB1*0407	0.0%	0.5%
DRB1*0408	1.0%	3.5%
DRB1*07	9.5%	7.0%
DRB1*08	1.0%	0.5%
DRB1*0901/902	3.0%	0.5%
DRB1*1001	3.0%	3.5%
DRB1*11	5.5%	6.5%
DRB1*12	0.5%	0.5%
DRB1*13	8.5%	6.0%
DRB1*14	1.5%	2.0%
DRB1*15	2.5%	11.0%
DRB1*16	4.5%	1.5%

b)

***HLA-DRB1* allele**	**German cohort**
DRB*01	16.3%
DRB*02	9.6%
DRB*03	6.7%
DRB*0401	20.8%
DRB*0402	3.9%
DRB*0404	3.4%
DRB*0405	3.9%
DRB*0407	3.9%
DRB*0408	0.6%
DRB*07	9.6%
DRB*08	2.2%
DRB*09	1.1%
DRB*10	1.7%
DRB*11	6.2%
DRB*12	2.8%
DRB*13	6.7%
DRB*14	0.6%

a) Allelic frequencies of HLA-DRB1 in French index patients. Each French family cohort consisted of 100 trio families. +rare HLA-DRB1 variants other than DRB*0101 and DRB*0102; b) Allelic frequencies of HLA-DRB1 in German patients (n=90)
