# Supplementary material for: Association of MICA with rheumatoid arthritis independent of known HLA-DRB1 risk alleles in a family-based and a case control study
Source: Arthritis Res Ther. 2009 May 1;11(3):R60. doi: 10.1186/ar2683 (PMC2714103; doi:10.1186/ar2683)
Supplement: Additional data file 2 — Background information for the conditional logistic regression method applied for family based analysis. [file ar2683-S2.pdf]

Additional data file 2: Methodological remarks

Data from case trio families are normally analysed using the standard TDT test. Here the analysis is complicated by the fact that the MICA SNP of interest is not in linkage equilibrium with the neighbouring HLA-DRB1 polymorphism that are already known to be related to RA. Thus a method is needed to investigate whether MICA has an independent contribution after adjusting for the known effect of the HLA-DRB1 polymorphism. We presuppose phased haplotype data.

The basic event to be modelled is the transmission of one of two competing haplotypes from parent to child. Note that the haplotypes include not only the allele of the SNP of interest but also the alleles of those polymorphisms for which we want to adjust. We assume – as with the TDT test – that transmission from the father and from the mother are independent events.

A statistical standard method to model binary data is logistic regression. In general let B be a binary random variable and z denote a vector of observed covariates. The logistic model reads:

$$pr(B=1|z)=\frac{\exp(\alpha+\beta^t z)}{1+\exp(\alpha+\beta^t z)} \quad \text{or equivalently}$$

$$\ln\left(\frac{pr(B=1|z)}{1-pr(B=1|z)}\right)=\alpha+\beta^t z$$

Given data, the coefficients α and β can be estimated by standard maximum likelihood methods.

As always one of the two parent's haplotypes is transmitted to the child in this application, the transmission probability is 50% when the competing haplotypes are identical. Thus α=0. The covariate z consists of a pair of vectors (z_1, z_2) characterizing the two haplotypes competing for transmission. Vector z_i consists of indicators for the alleles of all dichotomised polymorphisms in haplotype i. z_i=0

indicates the chosen reference haplotype. Assuming an additive model the probability of transmission only depends on differences in the competing haplotypes:

$$\ln\left(\frac{pr(transmission(haplotype1) \mid z_1, z_2)}{1 - pr(transmission(haplotype1) \mid z_1, z_2)}\right) = \beta^t (z_1 - z_2)$$

Estimated effect sizes are relative to the reference allele. If needed, this model can be extended to include also interaction terms. This particular model is also known as conditional logistic regression.
