# Supplementary material for: Association of MICA with rheumatoid arthritis independent of known HLA-DRB1 risk alleles in a family-based and a case control study
Source: Arthritis Res Ther. 2009 May 1;11(3):R60. doi: 10.1186/ar2683 (PMC2714103; doi:10.1186/ar2683)
Supplement: Additional data file 3 — A table providing detailed results of conditional logistic regression models of all French families. [file ar2683-S3.pdf]

Additional data file 3: Details of conditional logistic regression model of all French families when *MICA*-250 is either included (Model 1) or excluded (Model2):

**Model 1**

| Model Term | OR (95% CI) | p-value |
|---|---|---|
| S3P | 3.31 (2.17-5.16) | 1.68E-09 |
| S2 | 5.75 (3.44-10.06) | 1.21E-14 |

| Likelihood ratio: | degree of freedom | p-value |
|---|---|---|
| 82.8 | 2 | 4.25E-10 |

**Model 2**

| Model Term | OR (95% CI) | p-value |
|---|---|---|
| S3P | 3.69 (2.39-5.82) | 1.20E-10 |
| S2 | 5.65 (3.34-9.99) | 8.73E-14 |
| *MICA*-250A | 0.56 (0.38-0.83) | 2.81E-03 |

| Likelihood ratio: | degree of freedom | p-value |
|---|---|---|
| 92.4 | 3 | 6.31E-10 |

**Likelihood-Ratio-Test model 2 vs. Model 1.: p=0.002**

The odds ratio is for transmission of the indicated haplotype in comparison to the reference allele. For the *HLA-DRB1*-locus, allele L was used as reference. *HLA-DRB1* classification is according to Tezenas Du Montcel(7) (see Material and Methods).
