# Supplementary material for: Association of MICA with rheumatoid arthritis independent of known HLA-DRB1 risk alleles in a family-based and a case control study
Source: Arthritis Res Ther. 2009 May 1;11(3):R60. doi: 10.1186/ar2683 (PMC2714103; doi:10.1186/ar2683)
Supplement: Additional data file 4 — A table providing detailed results of logistic regression models of the German case control cohort. [file ar2683-S4.pdf]

Additional data file 4: Details of the logistic regression model of the German case/control cohort when MICA-250 is either included (Model 1) or excluded (Model2):

Model 1

Model Term	OR (95% CI)	p-value
S3P	2.1 (1.33-3.37)	1.26E-03
S2	3.52 (1.98-6.46)	6.00E-06

Likelihood ratio:	degree of freedom	p-value
58.4	3	4.18E-10

Model 2

Model Term	OR (95% CI)	p-value
S3P	2.14 (1.34-3.45)	0.001
S2	3.23 (1.81-5.92)	2.90E-05
MICA-250A	0.6 (0.37-0.96)	0.032

Likelihood ratio:	degree of freedom	p-value
63.7	4.0	6.93E-10

Likelihood-Ratio-Test model 2 vs. Model 1.: p=0.02

The odds ratio is for susceptibility to RA for the indicated alleles in an additive model. For the *HLA-DRB1*-locus, allele L was used as reference. *HLA-DRB1* classification is according to Tezenas Du Montcel(7) (see Material and Methods).
