# Supplementary material for: Association of MICA with rheumatoid arthritis independent of known HLA-DRB1 risk alleles in a family-based and a case control study
Source: Arthritis Res Ther. 2009 May 1;11(3):R60. doi: 10.1186/ar2683 (PMC2714103; doi:10.1186/ar2683)
Supplement: Additional data file 5 — A table providing a representation of association analysis in all informative families controlling for LD with DRB1. [file ar2683-S5.pdf]

Additional data file 5: Representation of association analysis in all informative families controlling for LD with DRB1

	Transmitted	Non-transmitted	OR (95%CI)
**Parents with DRB1 genotype S3P/S2**			
Haplotype S3P _*MICA*-250A	2	6	0.33 (0.02-5.11);
Haplotype S3P _*MICA*-250G (≙S3P-*MICA*-250A expected)	3	3	
**Parents with DRB1 genotype S2/L**			
Haplotype S2_*MICA*-250A	6	2	0.45 (0.04-6.76)
Haplotype S2_*MICA*-250G (≙S2-*MICA*-250A expected)	20	3	
**Parents with DRB1 genotype S3P/L**			
Haplotype S3P _*MICA*-250A	22	10	0.44 (0.04-2.73)
Haplotype S3P *MICA*-250G (≙S3P-*MICA*-250A expected)	10	2	
**Parents homozygous for DRB1 genotype**			
MICA-250A observed	21	42	0.50 (0.23-1.09)
MICA-250A expected	31.5	31.5	

HLA-DRB1 alleles are classified according to Tezenas DuMontcel (see Material and Methods). All parents included in this table are informative (=heterozygous) for *MICA*-250. OR: odds ratio of *MICA*-250A on transmission. 95%CI: 95% Confidence interval
